# CSF cytokine profile distinguishes multifocal motor neuropathy from progressive muscular atrophy

**DOI:** 10.1212/NXI.0000000000000138

**Published:** 2015-08-06

**Authors:** Takahiro Furukawa, Naoko Matsui, Koji Fujita, Hiroyuki Nodera, Fumitaka Shimizu, Katsuichi Miyamoto, Yukitoshi Takahashi, Takashi Kanda, Susumu Kusunoki, Yuishin Izumi, Ryuji Kaji

**Affiliations:** From the Department of Clinical Neuroscience (T.F., N.M., K.F., H.N., Y.I., R.K.), Institute of Health Biosciences, Tokushima University, Tokushima, Japan; Department of Neurology and Clinical Neuroscience (F.S., T.K.), Yamaguchi University Graduate School of Medicine, Ube, Japan; Department of Neurology (K.M., S.K.), Kinki University School of Medicine, Osaka, Japan; and National Epilepsy Center (Y.T.), Shizuoka Institute of Epilepsy and Neurological Disorders, Shizuoka, Japan.

## Abstract

**Objective:**

We aimed to compare the cytokine and chemokine profiles of patients with multifocal motor neuropathy (MMN) with those of patients with progressive muscular atrophy (PMA) and amyotrophic lateral sclerosis (ALS) to investigate immunologic differences in the CNS.

**Methods:**

CSF from 12 patients with MMN, 8 with PMA, 26 with sporadic ALS, and 10 with other noninflammatory neurologic disorders was analyzed for 27 cytokines and chemokines using the multiplex bead array assay. Cytokine titers of the 4 groups were compared, and correlations between the titers of relevant cytokines and clinical parameters were evaluated.

**Results:**

There were no obvious intrathecal changes except for interleukin (IL)-1 receptor antagonist in patients with MMN. In contrast, IL-4, IL-7, IL-17, eotaxin/CCL11, fibroblast growth factor-2 (FGF-2), granulocyte colony-stimulating factor (G-CSF), and platelet-derived growth factor BB titers were significantly elevated in patients with PMA and ALS; of these, FGF-2 and G-CSF titers were elevated compared with those in patients with MMN. IL-4 and IL-10 titers were high in patients with ALS, particularly patients with possible ALS presenting with a slowly progressive course or mild symptoms.

**Conclusions:**

The CSF cytokine profile of patients with MMN is distinct from that of patients with PMA and ALS. The similarity of the cytokine profiles between patients with PMA and ALS suggests that PMA shares common immunologic features with ALS in the CNS, even without clinical evidence of upper motor neuron involvement.

Multifocal motor neuropathy (MMN) is an immune-mediated neuropathy characterized by the lower motor neuron (LMN) syndrome, typically involving asymmetric muscle atrophy and weakness of the distal upper limbs.^1–3^ The early and accurate diagnosis of MMN is critical because immunotherapy, such as IV immunoglobulin, is often effective. The diagnostic features of MMN are conduction block (CB) in multiple peripheral nerves and anti-GM1 IgM antibodies.^4–6^ In cases lacking those features, however, MMN is often underdiagnosed^7–9^ or misdiagnosed as amyotrophic lateral sclerosis (ALS) or progressive muscular atrophy (PMA), a pure LMN variant of motor neuron disease.^10^ Conversely, ALS and PMA may be misdiagnosed as MMN because ALS may lack apparent upper motor neuron (UMN) signs^11,12^ and PMA by definition does not present any UMN signs during the whole course, even though it is suspected to be a form of ALS.^13,14^ It is important to distinguish MMN from PMA or ALS. We recently found that proinflammatory cytokines are elevated in the sera of patients with MMN, whereas few cytokine abnormalities are observed in the sera of patients with ALS.^15^ Instead, cytokine abnormalities have been reported in the CSF of patients with ALS, suggesting that CNS inflammation plays a crucial role.^16–18^ However, it is unclear whether CSF cytokine profiles differ between patients with MMN and patients with motor neuron disease (PMA and ALS) because of the paucity of data for MMN and PMA. To address this issue, we evaluated multiple cytokine/chemokine levels in the CSF of patients with MMN, PMA, and ALS.

## METHODS

### Patients.

We conducted a retrospective case-control study. The diagnosis of MMN was based on the diagnostic categories proposed by the European Federation of Neurological Societies and the Peripheral Nerve Society.^19^ We defined PMA according to the criteria described previously^13^: (1) diagnosed within 5 years; (2) clinical and electrophysiologic evidence of LMN involvement in 2 or more of 4 regions (bulbar, cervical, thoracic, and lumbosacral); (3) no CB in nerve conduction studies; and (4) no clinical UMN signs and symptoms. The diagnosis of ALS was made using the revised El Escorial criteria; patients fulfilling the “clinically definite,” “clinically probable,” “clinically probable—laboratory-supported,” or “possible” criteria were diagnosed with ALS.^20^ CSF samples were obtained from 10 patients with other noninflammatory neurologic disorders (ONDs), 12 with MMN (untreated), 8 with PMA, and 26 with ALS. All samples were immediately stored at −80°C until analysis. We determined sex, age, disease duration (time from symptom onset to CSF sampling), revised ALS Functional Rating Scale (ALSFRS-R) score,^21^ and disease progression rate (ΔALSFRS-R). ΔALSFRS-R was defined as [(ALSFRS-R full score – ALSFRS-R score at sampling)/disease duration expressed in months].^18^ Electrophysiologic studies were performed with commercially available EMG machines. Unilateral median, ulnar, tibial, and additional nerves when involvement was clinically suspected (e.g., radial, deep peroneal nerves) for motor studies, F-waves, and median, ulnar, and sural nerves for sensory studies were tested. CB was defined as a reduction in compound muscle action potential amplitude/area of >50% from distal to proximal stimulation in the absence of abnormal temporal dispersion.^19^ The presence of IgM and IgG antibodies against GM1, GM2, GD1a, GD1b, GM1b, GT1a, GT1b, GQ1b, GalNac-GD1a, and sulfate-3-glucuronyl paragloboside was tested in patients with MMN by conventional ELISA at Kinki University.^22^

### Standard protocol approvals, registrations, and patient consents.

The procedures followed were in accordance with the Helsinki Declaration of 1975, as revised in 1983. This study was approved by the Ethics Committee of the Tokushima University Hospital. All participants gave written informed consent.

### Cytokine and chemokine assays.

We performed multiplex bead array assay of serum and CSF samples using the Bio-Plex Pro Human Cytokine 27-plex Assay (Bio-Plex, Hercules, CA), as described previously.^23^ The panel was comprised of interleukin (IL)-1β; IL-1 receptor antagonist (IL-1ra); IL-2; IL-4; IL-5; IL-6; IL-7; IL-8/CXCL8; IL-9; IL-10; IL-12 (p70); IL-13; IL-15; IL-17; eotaxin/CCL11; fibroblast growth factor-2 (FGF-2); granulocyte colony-stimulating factor (G-CSF); granulocyte-macrophage colony-stimulating factor (GM-CSF); interferon (IFN)-γ; IFN-γ–induced protein 10 (IP-10)/CXCL10; monocyte chemotactic protein-1 (MCP-1)/CCL2; macrophage inflammatory protein (MIP) 1α/CCL3; MIP-1β/CCL4, platelet-derived growth factor BB (PDGF-BB); regulated on activation, normal T cell expressed and secreted/CCL5; tumor necrosis factor α (TNF-α); and vascular endothelial growth factor (VEGF). Soluble TNF receptor (TNFR1) was determined with an ELISA kit (BMS03; Cosmo Bio, Tokyo, Japan).

### Statistical analysis.

Differences in cytokine levels among the 4 groups (ONDs, MMN, PMA, and ALS) were compared by the Kruskal-Wallis test followed by Dunn multiple comparison post hoc analysis. Subsequently, differences in cytokine levels among the subgroups (MMN, PMA, possible ALS, and ALS except possible) were also analyzed. Differences in clinical data were assessed with Kruskal-Wallis, χ^2^, Student *t*, and Mann-Whitney *U* tests. Correlations between cytokine levels and clinical parameters (age, disease duration, ALSFRS-R score, and ΔALSFRS-R) were assessed with Spearman rank correlation test. *p* values <0.05 were considered statistically significant. The data were analyzed using GraphPad Prism5 and SPSS20.

## RESULTS

### Clinical manifestations.

We enrolled 12 patients with MMN (12 male; age 41.1 ± 16.4 years), 8 with PMA (7 male; age 67.1 ± 9.4 years), and 26 with ALS (15 male; age 65.0 ± 8.9 years). The diagnosis of ALS was definite in 7, probable in 6, probable—laboratory-supported in 5, and possible in 8. Region of ALS onset was bulbar in 7, trunk in 3, upper limbs in 10, and lower limbs in 6. We enrolled 10 patients with ONDs (9 male; age 70.3 ± 11.4 years) as controls: 7 had idiopathic normal pressure hydrocephalus, 2 had myalgia/neuralgia, and 1 had tension headache. Of the 12 patients with MMN, 7 (58.3%) showed CB and 2 (16.7%) had anti-GM1 IgM antibodies. Other clinical and CSF findings are listed in table 1.

**Table 1:**
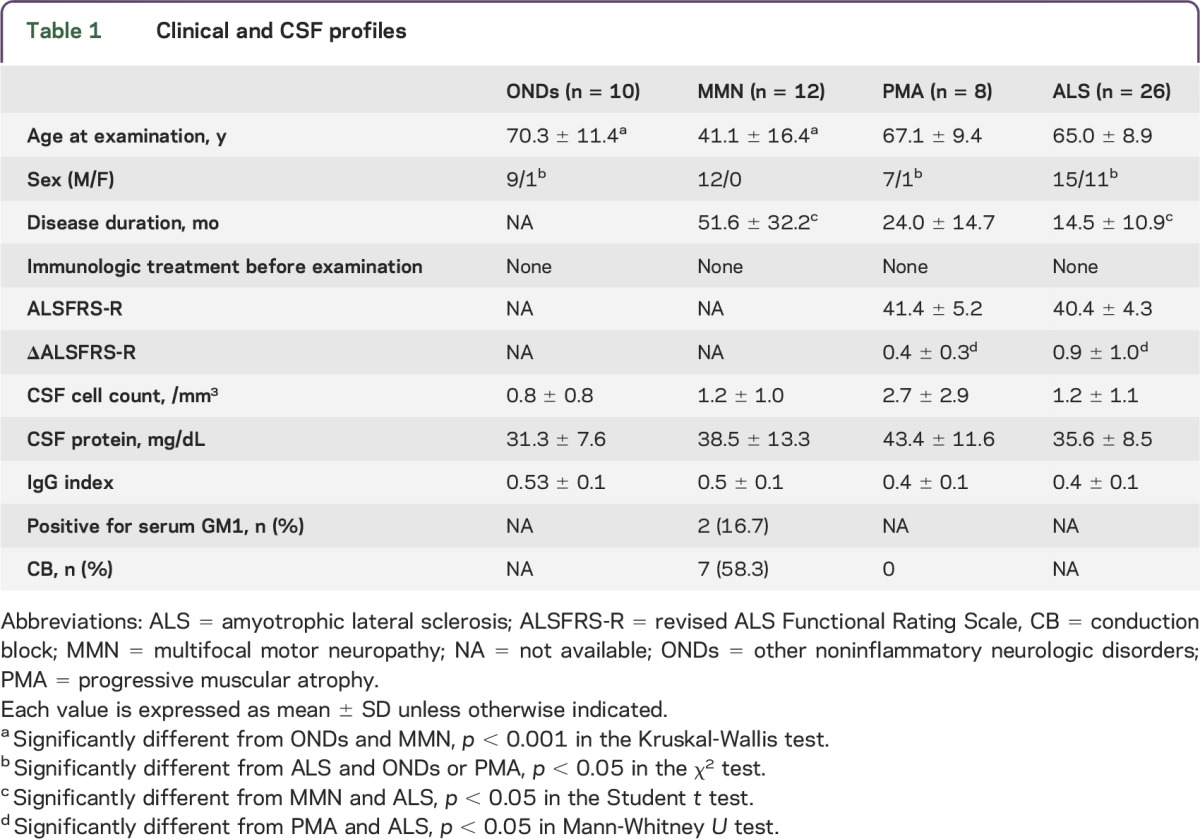
Clinical and CSF profiles

We compared the clinical profiles of patients with PMA with those of patients with ALS. The disease duration was longer in patients with PMA than in patients with ALS. Although ALSFRS-R scores did not differ (table 1), ΔALSFRS-R was lower in patients with PMA than in patients with ALS (*p* < 0.05). Region of PMA onset was upper limbs in 5 patients and lower limbs in 3. Four of the 8 patients with PMA required respiratory or nutrition support and 2 died of respiratory failure. Six patients with PMA received IV immunoglobulin (after CSF sampling) because the diagnosis of MMN without CB could not be excluded in the early stages; however, none responded to the treatment. None of the patients presented with UMN signs in the follow-up period, and all of them presented with progressive courses (observation time 54.8 ± 29.9 months). Overall, although none of the patients with PMA met the diagnostic criteria for ALS, the clinical features suggested that they presented with a variant phenotype of ALS (table 2). We also assessed the clinical profiles of patients with possible ALS and patients with probable/definite ALS. Patients with possible ALS showed slightly higher ALSFRS-R scores (43.6 ± 2.9) and lower ΔALSFRS-R (0.45 ± 0.22) than patients with probable/definite ALS (ALSFRS-R 40.4 ± 4.3; ΔALSFRS-R 0.76 ± 0.84), but there were no significant differences between them.

**Table 2:**
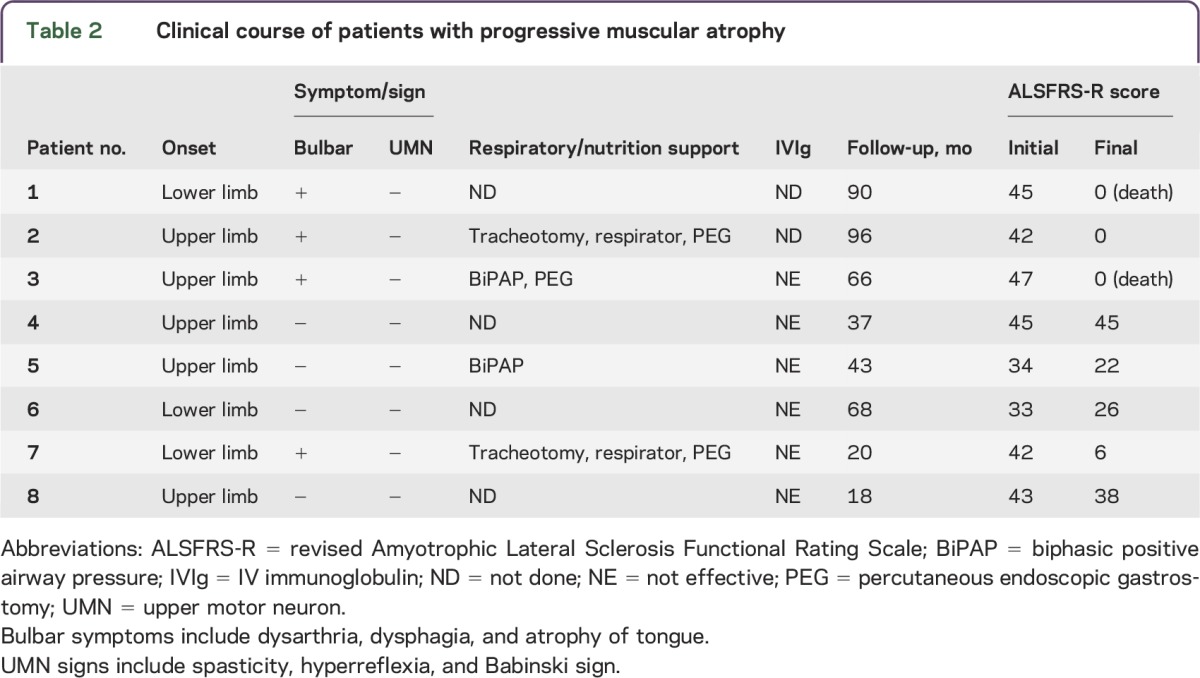
Clinical course of patients with progressive muscular atrophy

### CSF cytokine profiles.

**Figure 1:**
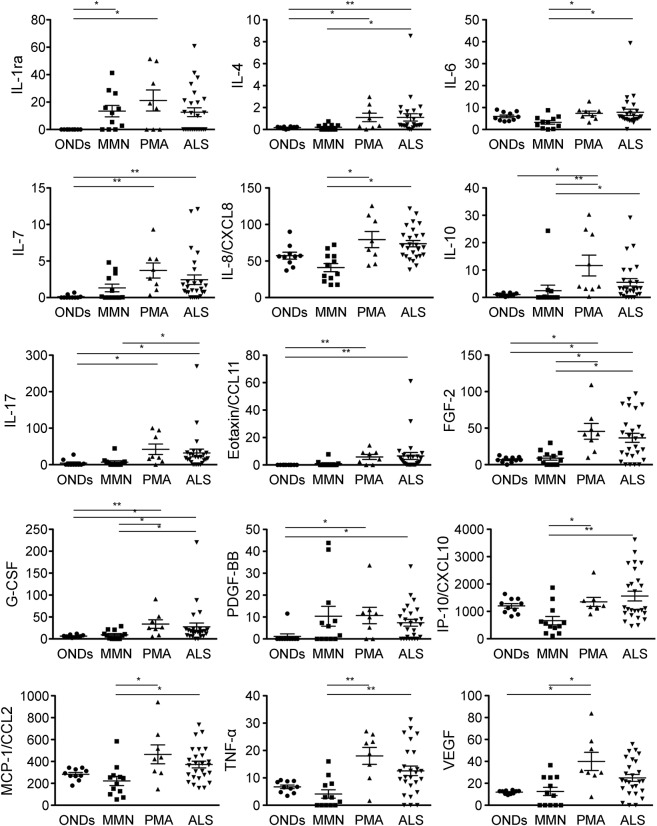
Cytokine titers determined by the multiplex bead assay Plotted are cytokine titers (pg/mL) in CSF of patients with other noninflammatory neurologic diseases (ONDs, n = 10), multifocal motor neuropathy (MMN, n = 12), progressive muscular atrophy (PMA, n = 8), and amyotrophic lateral sclerosis (ALS, n = 26).*p < 0.05, **p < 0.01. FGF-2 = fibroblast growth factor-2; G-CSF = granulocyte colony-stimulating factor; IL = interleukin; IL-1ra = IL-1 receptor antagonist; IP-10 = interferon-γ–induced protein 10; MCP-1 = monocyte chemotactic protein-1; PDGF-BB = platelet-derived growth factor BB; TNF-α = tumor necrosis factor α; VEGF = vascular endothelial growth factor.

IL-1ra was the only cytokine whose level was increased in patients with MMN compared with patients with ONDs. In contrast, as many as 10 cytokines/chemokines showed higher levels in patients with PMA than patients with ONDs: IL-1ra, IL-7, IL-10, IL-17, eotaxin/CCL11, FGF-2, G-CSF, PDGF-BB, VEGF, and TNFR1. Of these, IL-10, FGF-2, G-CSF, and VEGF were markedly elevated compared with patients with MMN as well (table 3; figure 1). IL-4, IL-7, IL-17, eotaxin/CCL11, FGF-2, G-CSF, and PDGF-BB levels were higher in patients with ALS than patients with ONDs; of these, IL-4, IL-17, FGF-2, and G-CSF levels were elevated compared with patients with MMN as well (table 3; figure 1). Both patients with PMA and patients with ALS had elevated levels of IL-7, IL-17, eotaxin/CCL11, FGF-2, G-CSF, and PDGF-BB. CSF cytokine profiles were different between patients with MMN and patients with motor neuron disease but were similar between patients with the 2 motor neuron diseases (PMA and ALS).

**Table 3:**
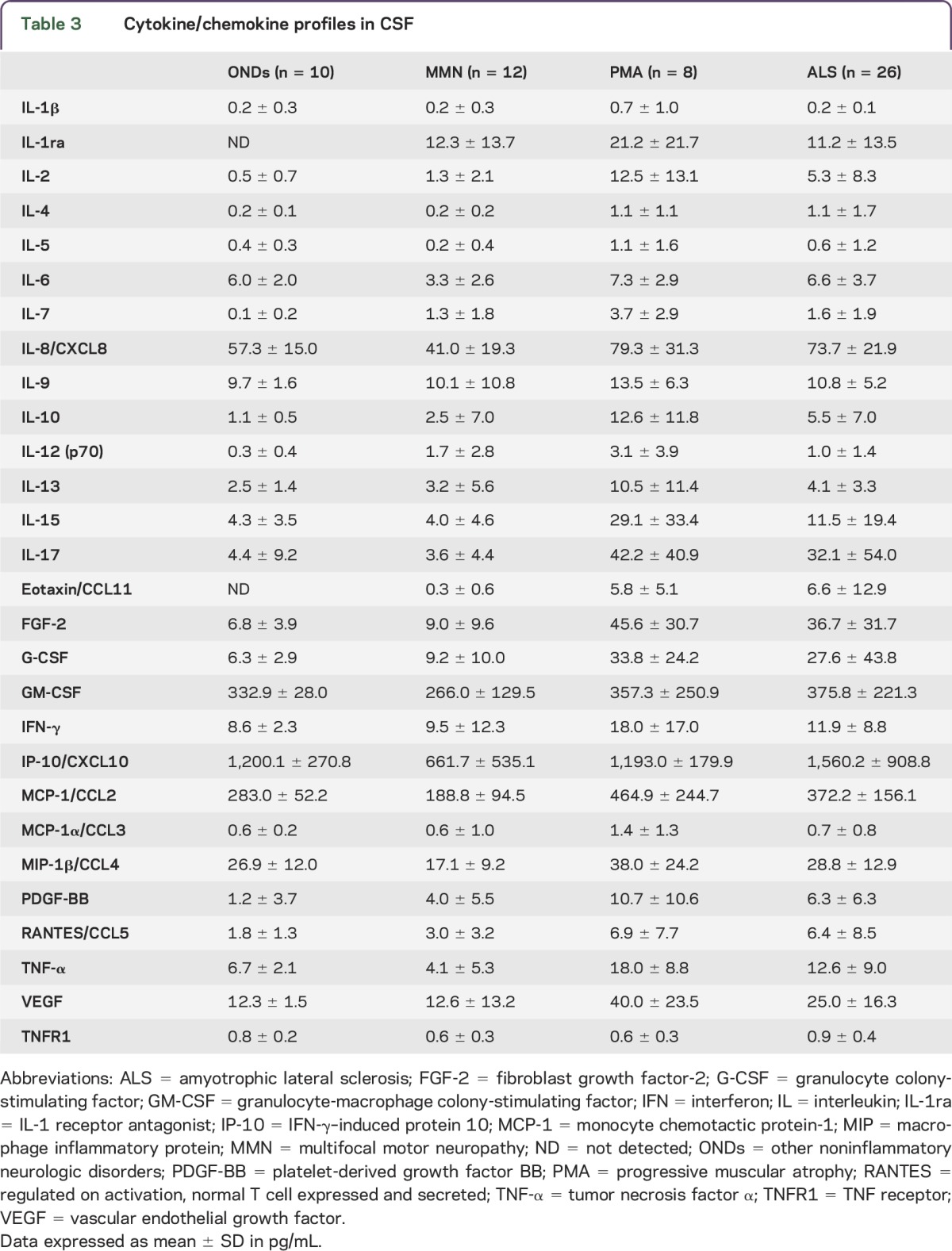
Cytokine/chemokine profiles in CSF

**Figure 2:**
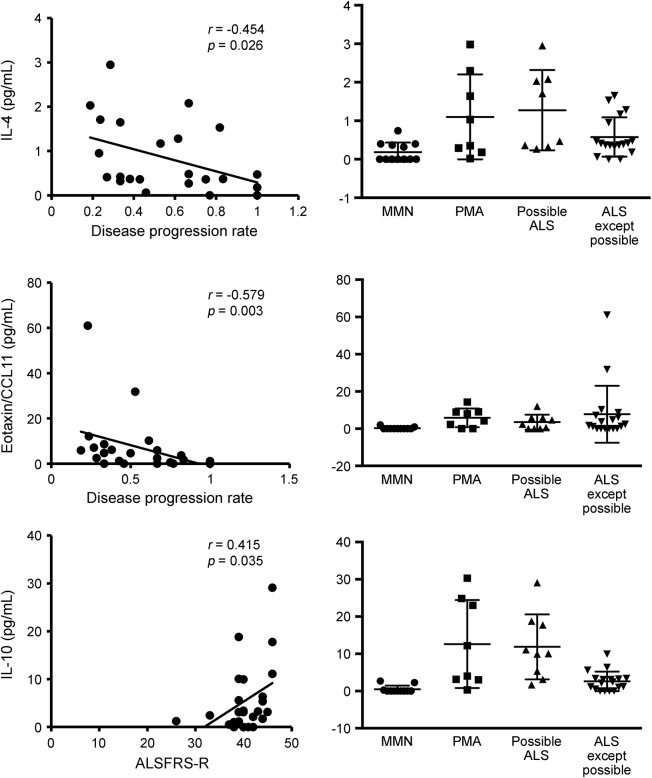
Correlation analysis and representative cytokine titers Left column shows correlations between cytokine levels in CSF and clinical parameters. Right column shows CSF cytokine titers (pg/mL) in multifocal motor neuropathy (MMN, n = 12), progressive muscular atrophy (PMA, n = 8), possible amyotrophic lateral sclerosis (ALS, n = 8), and ALS except possible ALS (n = 18). ALSFRS-R = revised Amyotrophic Lateral Sclerosis Functional Rating Scale; IL = interleukin.

IL-10 level was elevated in patients with ALS with high ALSFRS-R scores (*r* = 0.415, *p* = 0.035), indicating that elevation of IL-10 is associated with milder symptoms of the disease. IL-4 and eotaxin/CCL11 levels were higher in patients with ALS with lower ΔALSFRS-R (*r* = −0.454, *p* = 0.026 and *r* = −0.579, *p* = 0.003, respectively), indicating that those cytokines are found at higher levels in patients with slower disease progression (figure 2). In the subgroup analysis, cytokine patterns were not significantly different between patients with PMA and patients with possible ALS, but IL-4 and IL-10 levels tended to be higher in patients with PMA and patients with possible ALS than in patients with probable/definite ALS; however, this was not statistically significant (figure 2).

## DISCUSSION

Our results indicate that CSF inflammatory features are different between patients with MMN and those with motor neuron disease (PMA and ALS) but are similar between patients with motor neuron disease (PMA and ALS). In particular, FGF-2 and G-CSF levels were elevated in both patients with PMA and patients with ALS compared with patients with MMN and ONDs. Our epidemiologic study suggests that MMN is underdiagnosed because it is often misdiagnosed as “ALS without overt UMN signs.”^7^ Thus, it is critical to distinguish MMN from motor neuron diseases. This study included some patients with MMN who did not present with CB or with anti-GM1 IgM antibodies. However, the number of patients with MMN lacking CB and anti-GM1 IgM antibodies was too low to evaluate whether those cases showed a different cytokine profile from PMA and ALS. We have reported that proinflammatory cytokines, such as IL-1ra, IL-2, G-CSF, TNF-α, and TNFR1, are elevated in the sera of patients with MMN.^15^ In contrast, in the present study patients with MMN presented with abnormality only in CSF IL-1ra levels. Because IL-1ra is important for preventing ischemic or toxic brain injury in animal models,^24^ the elevated IL-1ra levels can be interpreted as a common regulatory mechanism to limit neuronal damage in MMN as well as ALS. In addition, CSF IL-1ra levels are elevated in patients with MMN with high serum IL-1ra levels (data not shown). Taken together, these findings indicate that cytokine abnormalities in MMN could be principally peripheral events. Although MMN may clinically resemble PMA and ALS, the CSF cytokine/chemokine profile in patients with MMN is quite distinct from that in patients with PMA and ALS. In our previous study,^15^ we also found that the levels of several proinflammatory cytokines were increased in the sera of patients with MMN, whereas no significant changes (except for IL-1ra level) were detected in the sera of patients with ALS. Therefore, the cytokine profiles in CSF complement rather than substitute for those in sera as useful biomarkers for MMN and motor neuron diseases.

IL-6, IL-8/CXCL8, IP-10/CXCL10, MCP-1/CCL2, and TNF-α levels were significantly higher in patients with PMA than patients with MMN, but not patients with ONDs. However, the levels of those cytokines (except IL-6) increased with age (data not shown), consistent with a previous study.^25^ Besides, aging itself might be associated with altered neuroinflammation.^26^ Therefore, the high levels of the 4 cytokines in patients with PMA could be associated with patient age, as patients in the MMN group were younger than those in the other groups.

In ALS mouse models with mutant Cu/Zn superoxide dismutase (SOD1), both beneficial (so-called M2 microglia, regulatory T cells, and Th2 cells) and deleterious (so-called M1 microglia and Th1 cells) immune responses can influence disease progression.^27^ We showed that IL-10 elevation was correlated with milder symptoms and that IL-4 and eotaxin/CCL11 elevation was correlated with slower disease progression in patients with ALS, suggesting that those cytokines may confer neuroprotection against ALS. Besides, subsequent analysis revealed that IL-4 and IL-10 tended to be higher in patients with PMA and possible ALS than in patients with probable/definite ALS, indicating slightly more severe symptoms in the latter. IL-10 has pleiotropic effects on immunoregulation.^28^ IL-4 is a Th2-associated cytokine.^27^ Eotaxin/CCL11 is a potent eosinophil chemoattractant cytokine associated with allergic airway inflammation.^29^ Thus, our results are consistent with the findings in SOD1 mouse models, in which regulatory T and Th2 cells can be beneficial.^27^ Furthermore, G-CSF was elevated in patients with motor neuron disease (PMA and ALS) compared with patients with MMN and disease controls. G-CSF can exert anti-apoptotic or anti-inflammatory effects as well as promote neurogenesis and angiogenesis in the CNS.^30,31^

Our results highlight the similarity in cytokine profiles between patients with PMA and patients with ALS. Both groups of patients have elevated levels of IL-7, IL-17, eotaxin/CCL11, FGF-2, G-CSF, and VEGF. In line with previous studies, patients with ALS also revealed abnormalities in the levels of such cytokines/chemokines as IL-7, IL-17, eotaxin/CCL11, FGF-2, G-CSF, and VEGF.^16–18^ IL-7 is a growth and differentiation factor for precursor B cells and plays a role in T cell activation.^32^ It is proposed that IL-17 contributes to several neurologic diseases, such as multiple sclerosis and neuromyelitis optica.^33,34^ FGF-2 deficiency prolonged survival and improved motor performance in the ALS mouse model,^35^ whereas FGF-2 was elevated in the present patients with PMA. The elevation of IL-7, IL-17, and FGF-2 may contribute to the CNS inflammatory process in patients with PMA and patients with ALS. Abnormalities in cytokine signaling have also been reported in other motor neuron diseases, such as spinal and bulbar muscular atrophy. In that disease, signaling of transforming growth factor β, a Th1-associated anti-inflammatory cytokine, is disrupted due to transcriptional dysregulation of its receptor, which is associated with polyglutamine-induced motor neuron damage.^36^ However, cytokines relevant to transforming growth factor β, such as IL-12 or IFN-γ, did not show abnormal levels in patients with PMA and ALS in the present study.

There is ongoing debate about whether PMA is a distinct disease entity or one spectrum of ALS. The current criteria for ALS diagnosis are based on the distribution of both UMN and LMN signs in various parts of the body, and thus “clinically pure LMN disease,” or PMA, is not classified as ALS. In fact, besides the UMN signs, PMA and ALS have somewhat different clinical characteristics. In the present study, the patients with PMA were mostly male and rarely had onset in the bulbar region, as previously reported.^13,14^ Nonetheless, several studies of clinical PMA cases revealed UMN abnormalities at autopsy.^37–39^ Moreover, a recent study reported that TAR DNA-binding protein 43 kDa pathology in the motor cortices or the hippocampus was common in both clinical ALS and PMA cases, suggesting that PMA is pathologically linked to ALS.^40^ The present study provides immunologic evidence that PMA and ALS are linked to each other.

## GLOSSARY

ALS: amyotrophic lateral sclerosis
ALSFRS-R: revised ALS Functional Rating Scale
CB: conduction block
FGF-2: fibroblast growth factor-2
G-CSF: granulocyte colony-stimulating factor
GM-CSF: granulocyte-macrophage colony-stimulating factor
IFN: interferon
IL: interleukin
IL-1ra: IL-1 receptor antagonist
IP-10: IFN-γ–induced protein 10
LMN: lower motor neuron
MCP-1: monocyte chemotactic protein-1
MIP: macrophage inflammatory protein
MMN: multifocal motor neuropathy
OND: other noninflammatory neurologic disorder
PDGF-BB: platelet-derived growth factor BB
PMA: progressive muscular atrophy
SOD1: Cu/Zn superoxide dismutase
TNF-α: tumor necrosis factor α
TNFR1: TNF receptor
UMN: upper motor neuron
VEGF: vascular endothelial growth factor
